# The α4 Nicotinic Acetylcholine Receptor Is Necessary for the Initiation of Organophosphate-Induced Neuronal Hyperexcitability

**DOI:** 10.3390/toxics12040263

**Published:** 2024-03-31

**Authors:** Peter M. Andrew, Wei Feng, Jonas J. Calsbeek, Shane P. Antrobus, Gennady A. Cherednychenko, Jeremy A. MacMahon, Pedro N. Bernardino, Xiuzhen Liu, Danielle J. Harvey, Pamela J. Lein, Isaac N. Pessah

**Affiliations:** 1 Department of Molecular Biosciences, UC Davis School of Veterinary Medicine, Davis, CA 95616, USA; pandrew@ucdavis.edu (P.M.A.); wfeng@ucdavis.edu (W.F.); jonas.calsbeek@gmail.com (J.J.C.); spantrobus@ucdavis.edu (S.P.A.); gche@ucdavis.edu (G.A.C.); jamacmahon@ucdavis.edu (J.A.M.); pnberna@ucdavis.edu (P.N.B.); xiuliu@ucdavis.edu (X.L.); 2 Department of Public Health Sciences, UC Davis School of Medicine, Davis, CA 95616, USA; djharvey@ucdavis.edu

**Keywords:** dihydro-β-erythroidine, diisopropylfluorophosphate, organophosphates, hippocampal slice cultures, mecamylamine, methyllycaconitine, neuronal hyperexcitability, nicotinic acetylcholine receptors (nAChRs), seizures

## Abstract

Acute intoxication with organophosphorus (OP) cholinesterase inhibitors can produce seizures that rapidly progress to life-threatening status epilepticus. Significant research effort has been focused on investigating the involvement of muscarinic acetylcholine receptors (mAChRs) in OP-induced seizure activity. In contrast, there has been far less attention on nicotinic AChRs (nAChRs) in this context. Here, we address this data gap using a combination of in vitro and in vivo models. Pharmacological antagonism and genetic deletion of α4, but not α7, nAChR subunits prevented or significantly attenuated OP-induced electrical spike activity in acute hippocampal slices and seizure activity in mice, indicating that α4 nAChR activation is necessary for neuronal hyperexcitability triggered by acute OP exposures. These findings not only suggest that therapeutic strategies for inhibiting the α4 nAChR subunit warrant further investigation as prophylactic and immediate treatments for acute OP-induced seizures, but also provide mechanistic insight into the role of the nicotinic cholinergic system in seizure generation.

## 1. Introduction

Organophosphorus cholinesterase inhibitors (OPs) are a class of toxic chemicals widely used as insecticides that have also been weaponized for use against military and civilian targets [1,2]. OPs pose a significant risk to public health and national security [2,3,4]. OP insecticides account for over 100,000 annual deaths globally, primarily as the result of accidental exposures or suicidal self-poisonings [5,6]. In addition, there have been numerous high-profile instances of malicious OP nerve agent use, including the 1995 Tokyo subway sarin gas attack, more recent civilian exposures in the Syrian Civil War, and targeted assassination attempts on North Korean and Russian dissidents [2].

The canonical mechanism of acute OP neurotoxicity involves the inhibition of acetylcholinesterase (AChE; EC 3.1.1.7), which results in the hyperstimulation of acetylcholine receptors (AChRs) in the peripheral and central nervous systems [7]. Acute inhibition of AChE by >60–70% causes a “cholinergic crisis”, a clinical toxidrome characterized by muscle fasciculations and weakness, parasympathomimetic signs, the depression of respiratory control centers in the brainstem, and seizures that can progress to life-threatening status epilepticus (SE) [8,9]. The current standard of care for cholinergic crises includes atropine to inhibit muscarinic acetylcholine receptors (mAChRs), oximes to reactivate AChE that has not yet aged, and benzodiazepines for seizure management [10]. While prompt therapeutic intervention improves survival following acute OP intoxication, survivors often develop significant long-term morbidity, including progressive brain damage, electroencephalographic abnormalities, and cognitive impairment [6,10,11,12,13,14,15,16,17,18]. The severity and extent of chronic neurotoxicity observed following acute OP intoxication is proportional to the duration and intensity of the acute OP-induced SE [15,19,20]. Thus, a reasonable therapeutic strategy for mitigating adverse neurological sequelae following acute OP intoxication is to prevent the onset or minimize the duration and/or severity of OP-induced SE. However, identifying more effective therapeutic strategies has been stymied by the lack of a comprehensive understanding of the mechanisms involved in seizure generation following acute OP intoxication.

Most mechanistic studies of OP-induced seizures have focused on the importance of mAChR signaling in seizure initiation and the rapid transition to excessive glutamatergic signaling [7,21,22] with subsequent downregulation of inhibitory GABA receptors [23,24] to maintain seizures. Consequently, to date, the development of therapeutic strategies for terminating OP-induced seizures has largely focused on the pharmacological antagonism of muscarinic and glutamatergic signaling and positive allosteric modulation of GABAergic signaling. In contrast, the role of nicotinic AChR (nAChR) signaling has not been extensively investigated as a therapeutic target in OP-induced seizures. This may be due in part to the fact that appropriate models and pharmacological agents for selectively targeting nAChRs have not been readily available until recently. For example, most in vitro model systems composed of primary neurons are derived from the developing brain, and developing neurons fail to express and properly target functional nAChRs to synaptic junctions and extra-junctional locations [25,26]. Here, we assessed the role of nAChRs in OP-induced SE by leveraging our observation that functional nAChRs are expressed in acute hippocampal slice cultures derived from adult mice, and by adapting our mouse model of acute intoxication with the OP diisopropylfluorophosphate (DFP) [27] to transgenic mice with a targeted deletion of genes encoding specific nAChR subunits.

## 2. Materials and Methods

### 2.1. Materials

DFP and all test pharmaceuticals were obtained from the same sources for the in vitro and in vivo studies. DFP was purchased from Sigma (St. Louis, MO, USA) and stored at −80 °C. The DFP’s purity of ≥90% was confirmed via nuclear magnetic resonance as previously described [28]. Atropine sulfate (AS), 2-pralidoxime (2-PAM), and paraoxon were purchased from Sigma and stored at room temperature or −20 °C. Manufacturer certificates of analysis indicated their purities to be as follows: AS (lot #BCBM6966V), >97%; 2-PAM (lot #MKCG3184), >99%; and paraoxon (#MFCD00007316), 99.9%. Nicotine was purchased from Fisher Scientific (Fisher Scientific; Fair Lawn, NJ, USA). Manufacturer certificates of analysis indicated that the purity of the nicotine (#AC181420050) was >99%. Mecamylamine hydrochloride was purchased from AK Scientific (Union City, CA, USA). Dihydro-β-erythroidine hydrobromide (DHβE) and methyllycaconitine citrate (MLA) were purchased from Tocris (Bristol, UK). Manufacturer certificates of analysis indicated their purities to be as follows: MEC (lot #TC23203), >98%; DHβE (batch #11A/249912), >98%; and MLA (batch #23A/251394), >98%.

### 2.2. Animals

Adult male WT C57BL/6J mice (8–10 weeks old) were obtained from The Jackson Laboratory (Sacramento, CA, USA) and allowed to acclimate for at least 7 d prior to surgical instrumentation for electroencephalography (EEG) measurements or experimentation. Three breeding pairs of heterozygous α4-nAChR global knockout (KO) mice on a C57BL/6J background [29] were provided by Henry A Lester, PhD, of the California Institute of Technology (Pasadena, CA, USA) and three breeding pairs of homozygous α7-nAChR global KO mice on a C57BL/6J background [30] (RRID #003232) were purchased from The Jackson Laboratory (Sacramento, CA, USA). These transgenic mice were bred at UC Davis to generate homozygous α4-nAChR KO mice and homozygous α7-nAChR KO mice that were used as adults for experimentation. The genotype of each animal used for experimentation was confirmed upon weaning at postnatal day 21 using the primers described in Table 1. Additional details for genotyping the α4-nAChR KO animals are provided in the Supplementary Materials. Homozygous α7-nAChR KO animals were genotyped using the protocol provided by The Jackson Laboratory (https://www.jax.org/Protocol?stockNumber=003232&protocolID=28408 accessed on 19 March 2024). 

All weaned and adult mice were group-housed (up to 4 mice per cage) in standard plastic shoebox cages in facilities fully accredited by AAALAC International under controlled environmental conditions (22 ± 2 °C, 40–50% humidity, 12:12 light–dark cycle). Animals were provided standard rodent chow (LabDiet #5058) and tap water ad libitum.

### 2.3. Primary Neuron–Glia Co-Cultures

Briefly, hippocampal or cortical neuronal/glial cultures were dissociated from C57BL/6J mice at embryonic day 18 (E18) or postnatal day (PND) 30–45 and from Sprague Dawley rat pups at PND 0–1. For these E18 and PND 0–1 cultures, brains from male and female pups were combined, whereas for the adult cultures, dissociated cortical cells were prepared only from the male mice using an established protocol [31]. Cortical cells were plated onto poly-L-lysine-coated, clear-bottomed, black-walled, 96-well imaging plates (BD Bioscience, Franklin Lakes, NJ, USA) at a density of 75,000 cells/well in complete Neurobasal medium (ThermoFisher, Waltham, MA, USA) supplemented with 0.5 mM L-glutamine, GS21 supplement (Cat No. GSM-3100; MTI-GlobalStem Products, ThermoFisher, Gaithersburg, MD, USA), 10 mM HEPES, and 5% fetal bovine serum (FBS) (Atlanta Biologicals, Norcross, GA, USA). The FBS concentration was reduced to 2.5% in the first day and halved at each change of the media. For microelectrode array assays, 110,000 cells/well were seeded in a 12-well Maestro plate (Axion BioSystems, Atlanta, GA, USA). Cytosine arabinoside (5 μM; Sigma Aldrich, St. Louis, MO, USA) was added to all cultures at 2 days in vitro and half of the media was changed every 3–4 days (the cytosine arabinoside concentration was halved at each change of the media). Neurons were incubated at 37 °C with 5% CO_2_ and 95% humidity for 7–16 days.

### 2.4. Measurement of Ca^2+^ in Primary Neuron–Glia Co-Cultures

In primary neuron–glia co-cultures, spontaneous calcium oscillations (SCOs) were measured using the FLIPR TETRA plate-reader system, as previously described [32,33,34,35,36,37]. The FLIPR TETRA plate-reader platform continuously measured and recorded intracellular Ca^2+^ transients from the 96 wells, simultaneously, and responses from each well were compared to their own baseline activity. The culture medium was replaced with prewarmed (37 °C) Locke’s buffer composed of (in mM) NaCl 154, KCl 5.6, CaCl_2_ 2.3, MgCl_2_ 1, HEPES 8.6, glucose 5.6, and glycine 0.0001, at pH 7.4, supplemented with 0.0005 mM Ca^2+^ fluorescence dye Fluo-4-AM (Sigma-Aldrich) and 0.5 mg/mL bovine serum albumin fraction V (Fisher Scientific). Plates were returned to the culture incubator for 1 h at 37 °C. Next, cells were gently washed with Locke’s buffer 3 times and permitted to equilibrate for 10–15 min. Baseline SCO recordings were taken for 10 min. Then, the programmable 96-channel pipetting robotic system added vehicle or test chemicals, either individually or in combination (e.g., ACh + DFP) to the wells. Changes in SCO patterns (frequency and amplitude) were monitored for an additional 20 min. ScreenWorks Peak Pro Software (Version 3.2; San Jose, CA, USA) was used to normalize the magnitude of SCOs based on (Fmax − Fmin)/Fmin, where Fmax was the peak for each spike, and Fmin was the baseline just prior to the spike. Kinetic Reduction Configuration was used to score the amplitude of SCOs. The total SCO frequency was scored using Peak Frequency (BPM) with Configure Peak Detection with the following settings: smooth width 1, fit width 3, slope threshold 0.01, amplitude threshold dynamic 7. The total SCO amplitude was scored using Average Peak Amplitude with Configure Peak Detection with the following settings: smooth width 1, fit width 3, slope threshold 0.01, and amplitude threshold dynamic 7.

### 2.5. Preparation of Acute Hippocampal Slices

Whole mouse brains dissected from adult PND 60–80 male mice immediately after cervical dislocation were placed in a Petri dish with an ice-cold sucrose cutting solution consisting of (in mM) sucrose 150, NaCl 40, KCl 4, NaH_2_PO_4_H_2_O 1.25, CaCl_2_2H_2_O 0.5, MgCl_2_6H_2_O 7, glucose 10, and NaHCO_3_ 26. Each brain was affixed with glue to the plate of a vibrating-blade microtome (VT1000S; Leica, Bannockburn, IL, USA). The plate was submerged and maintained in the ice-cold, carbogen-gassed (95% O_2_ + 5%CO_2_, medical grade) sucrose cutting solution. The horizontal cutting thickness was set at 400 µm. Two to three slices from the medial hippocampus of each hemisphere were transferred to a recovery chamber (UNICO Incubator, L-CU60; MFI Medical Equipment, San Diego, CA, USA) containing artificial cerebrospinal fluid (aCSF; composed of, in mM, NaCl 125, KCl 3.5, NaH_2_PO_4_H_2_O 1.2, CaCl_2_2H_2_O 2.4, MgCl_2_6H_2_O 1.3, glucose 25, and NaHCO_3_ 26) at 32 °C and constantly bubbled with carbogen. After a 60 min stabilization period, the slices were transferred onto pMEAs (60pMEA200/30iR-Ti; Multichannel Systems GmbH, Reutlingen, Germany).

### 2.6. Measurement of AChE Activity in Mouse Neuronal/Glial Cell Cultures and Hippocampal Slices

AChE activity was measured in hippocampal slices set up using the same protocol described above for setting up slices on pMEAs. After recovery, the hippocampal slices were grouped (4–6 slices/group/mouse) and transferred to a container with DFP at 0 (aCSF only, used as a control), 3, or 20 µM in aCSF/carbogen for 0, 5, 10, 15, 20, 30, 45, or 60 min at 32 °C. At the end of each incubation time, the slices were transferred to cryotubes and stored at −80 °C until AChE activity was measured.

Hippocampal slices or neuronal/glial cell cultures were homogenized in phosphate-buffered saline (PBS; NaCl 137 mM, KCl 2.7 mM, Na_2_HPO_4_ 10 mM, KH_2_PO_4_ 1.8 mM, pH 7.4) supplemented with 1 mM EDTA (pH 8.0) using a glass–Teflon homogenizer. The homogenate was centrifuged at 700× *g* at 4 °C for 10 min. The supernatant was collected and centrifuged at 100,000× *g* for 60 min. The pellet was resuspended in PBS/EDTA buffer and the protein concentration of the homogenate was measured using the Pierce BCA protein assay kit (Thermo Scientific, Waltham, MA, USA). AChE activity was measured using the Ellman assay with acetylthiocholine as a substrate and the indicator 5,5′-dithiobis-2-nitrobenzoic acid (DTNB), mixed with 10 µg hippocampal lysate protein per well in 96-well plates (Falcon) [38]. The O.D./min was continuously recorded at 405 nm over 30 min to measure the hydrolytic product 5-mercapto-2-nitrobenzoic acid using a Synergy H1 microplate reader (Biotek; Winooski, VT, USA). The O.D./min was converted into a catalytic rate using the Beer–Lambert law:

AChE activity (μmole/min) = {Slope (OD/min) × 2.5 × 10^−4^ (L) × 106 (μmole/mole)} {14,150 [L/(mole × cm)] × 0.125 cm}.

GraphPad Prism software (Version 8; San Diego, CA, USA) was used for data analysis and graphing. The IC_50_ and EC_50_ values were determined using nonlinear regression.

### 2.7. In Vitro pMEA Setup for Measuring Electrical Spike Activity (ESA)

The perforated dual MEA slice system (MEA2100; Multichannel Systems GmbH, Reutlingen, Germany) consisted of an interface board (IFB-C) with an integrated signal processor and head stage (HE2X60), equipped with integrated amplifiers, A/D converters, and stimulus generators. A dual-perfusion system operated with the UPPER and LOWER perfusion partners running in concert to securely keep the slice adhered to the pMEA throughout the recording and to maintain a continuous inflow of fresh oxygenated aCSF + reagent at the slice surface and a suction outflow of the exchanged bath waste through the adhered surface of the slice. Inflow was controlled using a D-Sub 9 interface, a PPS2 peristaltic perfusion pump (set at a rate of 2 mL/min) fed through a cannula PH01 with an integrated heating element and temperature sensor with a temperature controller interface (TC02 Multichannel Systems GmbH, Reutlingen, Germany), and a VC-8 Valve Controller (Harvard Apparatus, Hamden, CT, USA). The temperature in the pMEA chamber (60pMEA200/30iR-Ti; Multichannel Systems GmbH, Reutlingen, Germany) was maintained at 32 + 0.25 °C for all experiments. UPPER perfusion outflow was regulated through vacuum suction through a surface-positioned metal cannula with a beveled end (Warner Instruments, ST-1 L/R 64-1401; Hamden, CT, USA). LOWER inflow was driven by gravity and controlled through an IV Administration set with solution flow regulator (TrueCare Biomedix, TCBINF033G; South Miami, FL, USA). LOWER outflow was controlled with a PPS2 peristaltic perfusion pump at a rate of 0.2 mL/min. Prior to starting UPPER perfusion, LOWER perfusion was adjusted in order to (1) maintain continuous fresh aCSF/reagent exchange underneath the pMEA chamber; (2) provide gentile and constant suction to firmly contact the slice with the electrodes; and (3) steadily perfuse aCSF + reagent through the slice.

The ESA detected with the pMEAs was represented by spontaneous excitatory postsynaptic field potentials (fEPSPs), which were captured simultaneously from the 60 electrodes at a sampling rate of 10 kHz within each pMEA. Data from individual electrodes were analyzed using Multi Channel Suite software, version 2020 (Multichannel Systems GmbH, Reutlingen, Germany). For data acquisition, the Spike Detector of the MCS Experimenter was selected using the Manual Threshold with a defined Falling Edge of −20 mV and Rising Edge of 0 mV. Post data acquisition, the data were processed using the Spike Analyzer/MCS Analyzer. A bin width of spikes/min was obtained for the spike activity analysis. The baseline recording during the first 10 min of the experiment was defined as Epoch I (0–10 min), followed by Epoch II (10–20 min) through Epoch VI (50–60 min), or beyond in some cases. Since the placement of each slice on a pMEA was not performed with stereotactic registry among the experiments, each electrode served as an independent measurement, and its temporal changes in mean spikes/min were normalized to its own baseline period (Epoch I), which served as the control. Post-acquisition statistical analyses and plots of these analyses were conducted using OriginLab (OriginLab Corporation, Northampton, MA, USA, 2019b). The methods used for statistical tests and normality tests are indicated in the figures/tables or Supplementary Materials, including the use of Prism GraphPad (GraphPad Software, V10) and CorelDRAW 2020 (Corel Corp.; Ottawa, ON, Canada).

### 2.8. In Vivo DFP Dosing Paradigm

Sample-size calculations based on regression analyses of the preliminary data confirmed that a total of 4–5 animals per group would provide sufficient power (80+%) to detect robust effects (e.g., geometric mean ratios of 2.0+) under two-sided testing with α = 5%. Mice were injected s.c. with 9.5 mg/kg DFP in PBS (3.6 mM Na_2_HPO_4_, 1.4 mM NaH_2_PO_4_, 150 mM NaCl, pH 7.2) in the scruff of the back of the neck; vehicle controls were injected with an equal volume (0.1 mL) of PBS (s.c.). Both the DFP and vehicle control animals were injected 1 min later with AS (0.1 mg/kg in sterile saline, i.m.) and 2-PAM (25 mg/kg in sterile saline, i.m.) in the hind leg. Combined treatment with AS and 2-PAM is necessary to prevent excessive death from DFP-induced cholinergic crisis [39].

A subset of mice were randomly selected using a random number generator for treatment with one of various pharmacological antagonists of nAChRs 10 min before DFP injection: the non-selective nicotinic antagonist mecamylamine hydrochloride (MEC; 9.5 mg/kg in sterile saline; AK Scientific, Union City, CA, USA), the α4-selective nAChR antagonist dihydro-β-erythroidine hydrobromide (DhβE; 5 mg/kg in sterile saline; Tocris, Bristol, UK), or the α7-selective nAChR antagonist methyllycaconitine citrate (MLA; 5 mg/kg in sterile saline; Tocris). These pharmacological reagents were injected s.c. in the scruff of the back of the neck. Vehicle control mice were injected with an equal volume (10 mL/kg) of sterile saline.

### 2.9. Quantification of Seizure Behavior

Seizure behavior in mice was quantified using a modified Racine scale from 0 to 5, with 0 corresponding to no seizure behavior and 5 corresponding to the most severe behavior, as previously described [27]. Seizure severity was scored every 5 min for the first 120 min after DFP injection, and then every 20 min until 240 min post DFP injection. After the 4 h monitoring period, the mice were weighed and then injected with 1 mL dextrose (5% *v*/*v* in saline; s.c.) in the scruff of the back of the neck and singly housed with wet food. Mice were weighed daily and provided wet food and dextrose as needed until their body weight returned to the pre-DFP baseline level (typically within 4–7 d).

### 2.10. EEG Surgery and Recordings

A subset of animals was instrumented for cortical electroencephalographic (EEG) monitoring in accordance with the UC Davis Rodent Survival Surgery guidelines. Adult male WT, α4 KO, and α7 KO C57BL/6J mice (8 weeks old) were deeply anesthetized using continuous isoflurane inhalation (5% for induction, 1–3% for maintenance; VetOne) in medical-grade oxygen (0.5 L/min; Airgas, Radnor, PA, USA) and compressed air (1 L/min; Airgas). Animals were secured in a stereotaxic frame (Stoelting Co., Wood Dale, IL, USA), and the surgical site was shaved and cleaned three times with alternating betadine scrub (Purdue Products L.P., Stamford, CT, USA) and 70% *v*/*v* isopropyl alcohol (Fisher HealthCare, Pittsburgh, Pennsylvania). Sterile ophthalmic ointment (Altaire Pharmaceuticals, Northville, NY, USA) was applied to prevent eye dryness. Analgesics (meloxicam, 2 mg/kg, s.c.) and antibiotics (ampicillin, 40 mg/kg, s.c.) were administered preoperatively.

An incision was made along the midline of the skull from the eyes to the neck, and two stainless steel head mount screws (P1 Technologies, Roanoke, VA, USA) were implanted into the skull (2 mm anterior of bregma/−1.5 mm lateral of midline; 2 mm posterior of bregma/+1.5 mm lateral of midline). Head mount screws were connected to sterile HD-X02 telemetry devices (Data Sciences International (DSI), St. Paul, MN, USA) inserted subcutaneously along the flank of the animal. The screws were further secured with a cap of dental cement, and the incision site was sutured (Ethicon Inc., Raritan, NJ, USA). Analgesia and antibiotics were administered daily for 2 d post surgery. Animals were allowed to recover for 7–10 d before DFP exposure. Four of the forty mice instrumented for EEG monitoring died or were euthanized prior to experimentation.

On the day of experimentation, animals with telemetry implants were placed on PhysioTel Receivers (DSI) and baseline EEG recordings were obtained for ~1 h prior to DFP intoxication using Ponemah software, version 5.61 (DSI). Animals instrumented for EEG were injected using the same DFP and nAChR antagonist dosing paradigm as the non-instrumented animals, and EEG data were recorded for ~4 h post intoxication. The few animals treated with DHβE (*n* = 3 of 8) that died during the post-intoxication monitoring period were excluded from the subsequent EEG analyses.

### 2.11. EEG Analyses

EEG traces were analyzed by researchers blinded to the experimental group using NeuroScore software, V3.3.9317-1 (DSI). Prior to these analyses, traces were preprocessed using a 1 Hz high-pass filter to remove movement artifacts. The time windows of interest were the baseline (pre DFP), early-seizure (first hour post DFP intoxication), and late-seizure (1–4 h post DFP) periods.

EEG root-mean-squared (RMS) values are elevated during seizure activity [40]. RMS values for EEG traces were extracted over 1 s epochs for the entire recording period. Baseline, early-, and late-seizure RMS values were determined by averaging the RMS values for each 1 s epoch over the recording window to produce a single average value for each epoch. RMS values were normalized by dividing the early- and late-seizure RMS values by the baseline RMS values.

The EEG spike rate is another electrographic metric consistently elevated during seizure activity [41]. The spike rate provides information that is complementary to the RMS, capturing the frequency of abnormal coordinated discharges of neuron populations. For spike rate analysis, spike amplitude threshold criteria were determined for each animal and defined as an amplitude of greater than 4 times the standard deviation of the average baseline oscillatory amplitude. For each animal, the amplitude (greater than the mean amplitude + 4X standard deviation) and duration criteria (20–70 ms) were used to identify individual spikes across the entire recording period. Spike count data were then binned into 1 min epochs for the entire recording period. The baseline spike rate was calculated by averaging spike count data over the recording period prior to the administration of DFP. The spike rate over the early- and late-seizure periods was normalized to this baseline rate for data analyses and presentation.

Key outcomes of the statistical analyses of EEG data included the RMS, normalized RMS, and spike rate. For the RMS and spike rate, there were three time epochs of interest: baseline (pre DFP), early seizure (first hour post DFP exposure), and late seizure (1–4 h post DFP exposure). For the normalized RMS, there were two epochs chosen for analysis, early and late seizure, both relative to the baseline period. Due to the repeated measurements over time within each animal, mixed-effects models, including animal-specific random effects, with key factors of interest including groups (Veh/WT, MEC, α4 KO, α7 KO, DHβE, MLA) and time epochs, were used to evaluate group differences. Any interaction between the two factors was considered and the Akaike Information Criterion was used to determine the best model. All outcomes were transformed using the natural logarithm to better meet the assumption of the model; spike rate values were first shifted by 0.1 prior to taking the log-transformation to account for zeros in the data. Specific contrasts for the within-group changes (as well as differences in the changes between the different treatment groups and the Veh/WT group) were constructed and tested with a Wald test. The Benjamini–Hochberg false discovery rate (FDR) was used to account for multiple comparisons. The results are presented as geometric mean ratios (GMRs) between the time epochs within each group and between the treatment groups and vehicle/WT controls. Point estimates of the ratios and 95% confidence intervals are presented in the figures. When the confidence interval for the GMR includes 1, there is no statistical evidence of a difference between the groups. All analyses were performed using SAS software, version 9.4, and alpha was set at 0.05; all reported results remained significant after the FDR procedure.

## 3. Results

### 3.1. Primary Neuronal/Glial Cell Cultures from Neonatal Rodents Express AChE but Fail to Target Functional nAChRs

This study originated from observations made by the UC Davis CounterACT Center of Excellence, whose goals included the development of rapid throughput in vitro assays to identify novel therapeutic interventions against neuroactive threat agents. Table 2 summarizes the research conducted as part of that initiative.

Several neuron/glia co-culture models derived from postnatal day (PND) 0–1 mouse or rat hippocampi or neocortices were investigated for their ability to form mature neuronal networks that expressed cell-bound AChE and developed robust spontaneous calcium oscillation (SCO) patterns, which are regulated by the balance of excitation–inhibition, mediated by the activity of ionotropic and metabotropic glutamatergic and GABAergic receptors [36,37]. While SCO patterns in these primary neuronal models have been shown to be uniquely altered when exposed to pharmacological/toxicological chemicals that have distinct molecular mechanisms, they failed to respond to OPs (either DFP or paraoxon) at levels that inhibited ≥95% of their cellular AChE catalytic activity (Table 2). Cortical cultures established from embryonic day 18 mouse pups also failed to respond to OPs and, compared to similar cultures derived from PND 0–1 mouse pups, expressed lower AChE activity at 14–17 days in vitro. Our attempts to culture primary neuron–glia co-cultures dissociated from the adult mouse cortex failed to produce sufficiently viable cultures that could be used for functional screening using the FLIPER Tetra to measure the effects of chemical probes on SCOs. An important clue to explain this paradox came from the observation that primary neurons derived from the perinatal rodent brain developed robust responses to atropine but failed to develop responses to nicotine (Table 2), suggesting an obligatory role of one or more nAChR subtypes for eliciting responses to OPs.

### 3.2. DFP Increased Spontaneous Electrical Spike Activity (ESA) Proportionate to AChE Inhibition in Acute Hippocampal Slices Prepared from Adult Mouse Hippocampi

**Figure 1:**
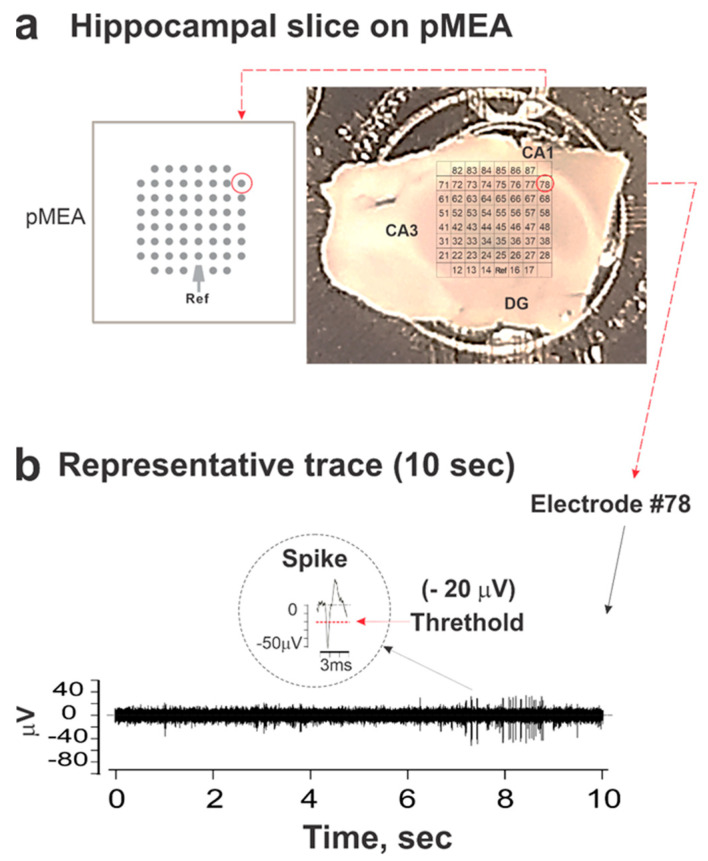
Recording of spontaneous network activity from mouse hippocampal slices on perforated microelectrode arrays (pMEAs). Mouse hippocampal slices were firmly secured on pMEAs using a dual-perfusion system. aCSF was perfused through each slice. (**a**) Although the slices were consistently placed on the 60 electrode arrays to record activity from the entire hippocampus, including CA1, CA3, and DG, the slices were not placed stereotactically. (**b**) A representative 10 s recording of spontaneous electrical spike activity (ESA) from electrode #78. Each spike event was identified as a signal with a Falling Edge that exceeded the threshold of −20 mV (red dashed line and arrow). A typical spike waveform of 3 ms is shown in the circle.

Mouse hippocampal slices were freshly prepared from adult male mice and randomly divided into two groups: one group that was exposed to either 3 or 20 µM DFP in aCSF for 0–60 min and then frozen for later AChE determinations, and a second group that was mounted on perforated microelectrode arrays (pMEAs) to record ESA before and after the perfusion of 0, 3, or 20 µM DFP. Figure 1a portrays the typical placement of a hippocampal slice on the pMEA. Once the slice was adhered to the pMEA and perfusion across the slice was adjusted, the recording of electrical activity revealed spontaneous ESA in the absence of external electrical stimulation. Slices recorded on pMEAs displayed spontaneous ESA that varied in basal frequency, although electrodes within or near CA1 typically produced the most active ESA (Figure 1b).

**Figure 2:**
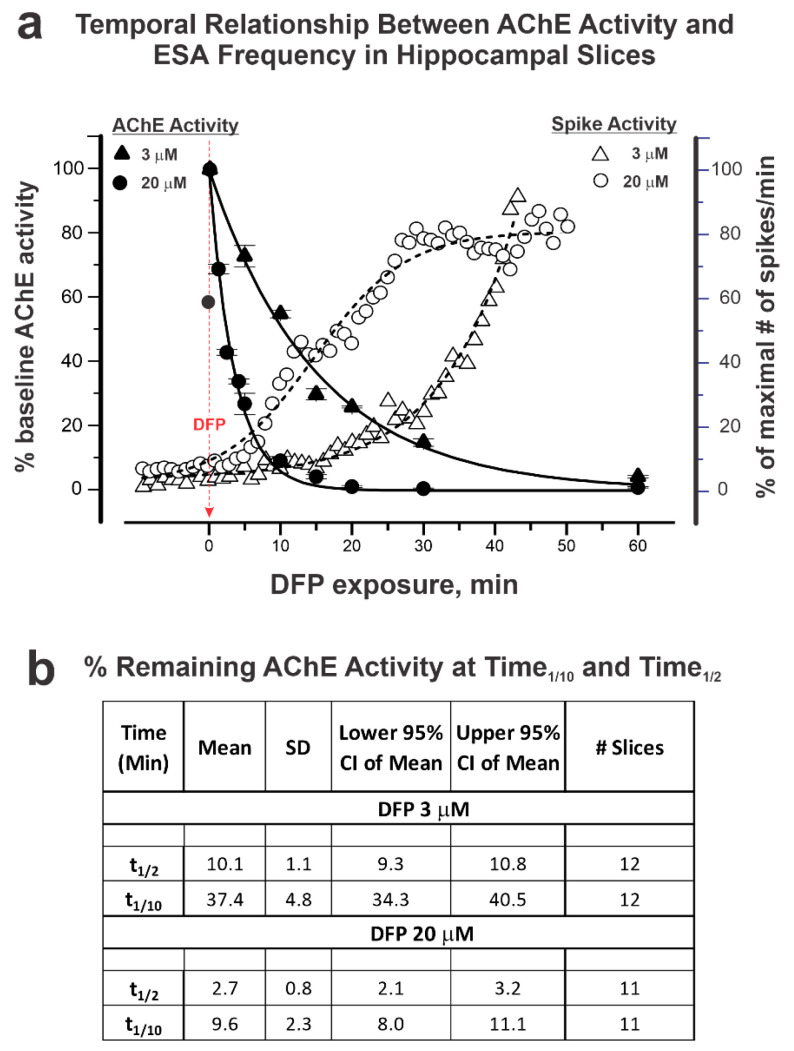
Temporal relationship between AChE activity and electrical spike activity (ESA) in hippocampal slices. AChE activity, measured using the Ellman assay, and ESA were assessed in acute hippocampal slices obtained from adult mice at varying times after the addition of DFP at 3 or 20 µM. (**a**) AChE activity (left y-axis; filled triangles and circles) and ESA (right y-axis; open triangles and circles), both shown as a % of the baseline activity prior to the addition of DFP. The baseline AChE activity range was 0.41–0.50 mmol/min/mg. Each time point represents the mean value of *n* = 5 independent AChE preparations, each performed in triplicate (totaling 11–12 slices from N = 28 mice). The ESA frequency (right y-axis) increased with exposure time and is presented as mean spikes/minute (% of maximum) from one representative slice exposed to 3 or 20 µM DFP (N = 24 and N = 17 active electrodes, respectively). (**b**) The times taken to achieve 50% and 90% inhibition of AChE (t1/2 and t1/10) were obtained by nonlinear regression fitting using OriginLab.

Figure 2a summarizes the relationship between the AChE catalytic activity and the length of time the slices were exposed to DFP (3 and 20 µM, filled triangle and filled circle, respectively). The rate with which DFP inhibited AChE was unexpectedly slow given the fact that the exposure paradigm consisted of through-slice perfusion of DFP. The addition of 3 and 20 µM DFP to the perfusate resulted in a 90% inhibition of AChE activity relative to the control aCSF within 37.4 ± 4.8 and 9.6 ± 2.3 min, respectively (Figure 2b). ESA frequency increased with DFP exposure time and was tightly correlated with the degree of AChE inhibition, which was highly dependent on the DFP concentration (Figure 2a,b). Importantly, perfusion with DFP did not significantly increase the ESA frequency until >80% of the slice AChE activity was inhibited, and only achieved peak ESA frequencies when >95% AChE was inhibited (Figure 2a).

**Figure 3:**
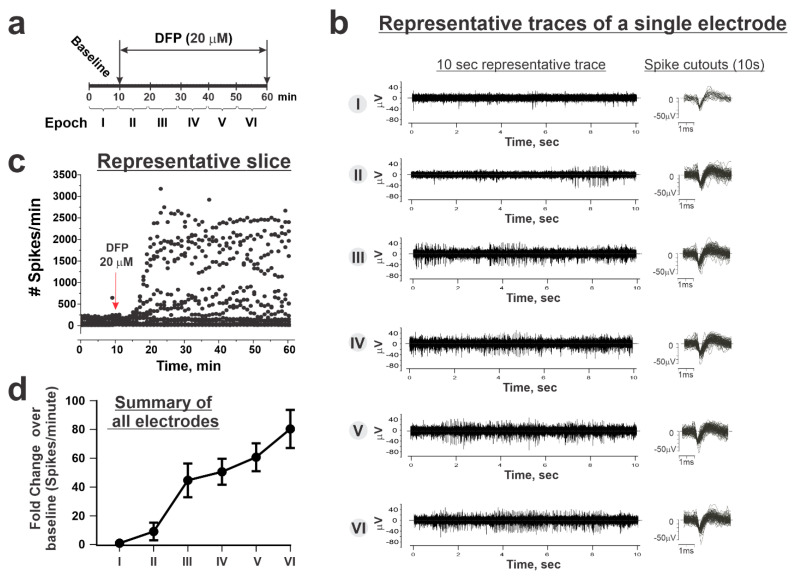
The temporal influence of DFP on hippocampal ESA. (**a**) Each slice recording was conducted over 60 min and analyses of ESA were subdivided into six consecutive epochs. The first 10 min served as the baseline and is defined as Epoch I. DFP (20 µM) was added to aCSF perfusate after the first 10 min (indicated by the red arrow in panel (**c**)), with subsequent 10 min recordings being defined as Epochs II through VI. (**b**) Representative 10 s recordings of ESA from a single electrode within each epoch. Spike cutouts of EPSP waveforms from each recording electrode were overlaid from each epoch. (**c**) ESA recorded from one slice from both active and inactive electrodes before and after the addition of 20 µM DFP into the perfusate. (**d**) Mean ESA ± 95% confidence interval (CI) from all active electrodes from all slices (N = 76 active electrodes from 10 slices, obtained from seven mice). The baseline recording (Epoch I) from each active electrode was used to normalize that electrode’s DFP response. Statistical analyses of these data are presented in Table 3.

Based on these results, all subsequent slice experiments were performed with 20 µM DFP in order to limit the experiments to a total of 60 min (10 min baseline + 50 min with DFP perfusion). Figure 3a illustrates the experimental protocol used for further evaluating the temporal influences of DFP on ESA. The 60 min experiment was divided into six epochs, at 10 min/epoch (Figure 3a). Epoch I recorded 10 min of the baseline period prior to the addition of DFP to the aCSF perfusate and served as the basis for normalizing ESA for each active electrode. Epochs II–VI were recorded subsequent to the addition of 20 µM DFP to the aCSF perfusate. Figure 3b shows a representative 10 s recording from each epoch and the overlay of all spike cutouts from these 10 sec recordings. Figure 3c shows the temporal changes in ESA frequency of all electrodes detecting activity in a single slice. Epochs II–VI from each active electrode were normalized to their respective baseline activity (Epoch I) and reported as fold changes in the spike rate over baseline (Figure 3d). Statistical analyses of normalized changes in the ESA frequency are summarized in Table 3. DFP (20 µM) produced a mean 9.1-fold increase in ESA frequency relative to baseline within the first 10 min (*p* = 0.039, Table 3), whereas within the last epoch (Epoch VI), DFP increased the ESA frequency 67.1–93.6-fold (lower–upper 95% of mean, Table 3).

#### 3.2.1. Mecamylamine Suppressed DFP-Triggered ESA Hyperexcitation

**Figure 4:**
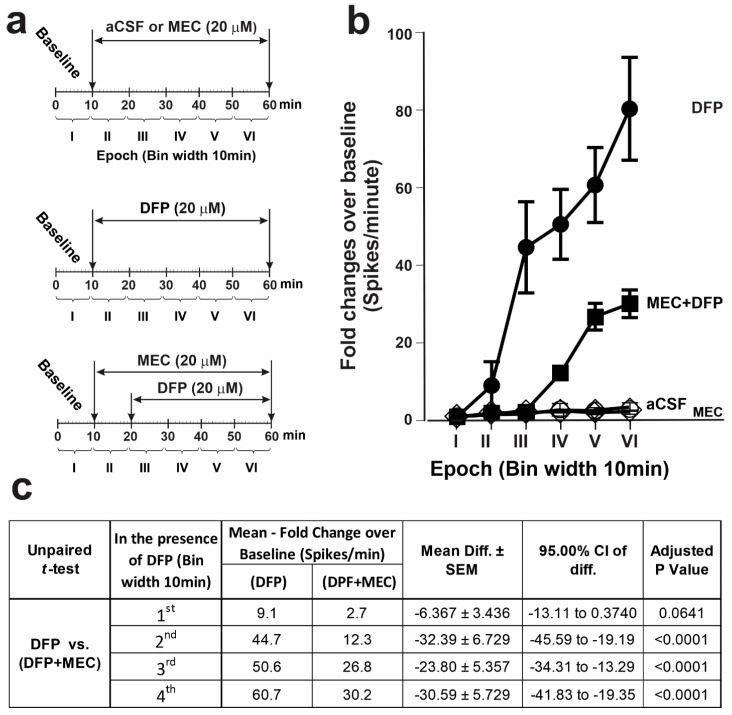
Mecamylamine (MEC) attenuated the DFP-triggered hyperactivity of hippocampal slices. (**a**) Schematic illustration of various exposure paradigms assessed for their effects on ESA. After a 10 min baseline recording, slices were perfused for an additional 50 min with either aCSF alone or aCSF to which MEC (20 µM) or DFP (20 µM) had been added. To determine whether MEC inhibited the effects of DFP on ESA, after 10 min of baseline recording, slices were perfused for 10 min with MEC (20 µM) and then with MEC and DFP (20 µM) for an additional 40 min. (**b**) Fold changes in the ESA relative to the baseline within each of the six epochs. Data shown as the mean ± 95% CI (aCSF (open circles), N = 28 electrodes from six slices obtained from six mice; the MEC-alone group (open diamonds), N = 23 electrodes from four slices obtained from four mice; the DFP-alone group (filled circles), N = 76 electrodes from 10 slices obtained from seven mice; the MEC + DFP group (filled squares; N = 56 electrodes from six slices obtained from six mice)). (**c**) Summary of the statistical analyses comparing ESA in the DFP group vs. the MEC + DFP group. Adjusted *p* values < 0.05 were regarded as significant.

Mecamylamine (MEC), a non-selective noncompetitive antagonist of nAChRs, was included in the aCSF perfusate in the absence or presence of DFP (Figure 4a). The perfusion of MEC in the absence of DFP had no significant influence on the ESA frequency compared to the perfusion of aCSF alone (Figure 4b). The addition of MEC to the perfusate at the beginning of Epoch II, 10 min prior to initiating DFP perfusion (20 µM) in Epoch III, dampened the ESA frequency compared to the perfusion of DFP alone during Epochs II–VI (Figure 4a,b). MEC suppressed DFP-triggered ESA frequencies 3.3-fold (*p* = 0.064) during the first 10 min. In contrast, during subsequent epochs, MEC suppressed the DFP-triggered ESA frequency to an even greater extent (Figure 4b,c).

#### 3.2.2. Dihydro-β-Erythroidine (DHβE) Reversibly Attenuated the DFP-Triggered Increases in ESA

**Figure 5:**
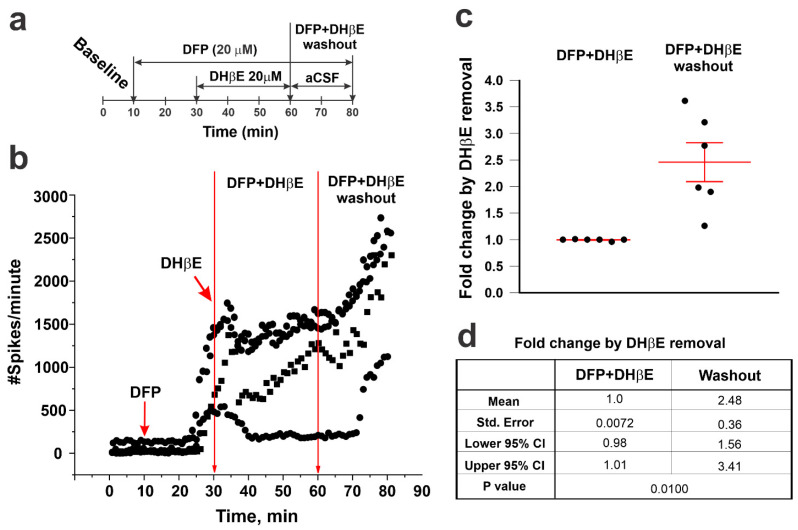
Selective α4 nAChR antagonism reversibly attenuated DFP-triggered hyperactivity. (**a**) Schematic of the exposure protocol: following a 10 min baseline recording, acute hippocampal slices were sequentially perfused with DFP (20 µM), the selective α4 nAChR antagonist, DHβE (20 µM), and then aCSF alone to remove DFP and DHβE. DHβE was introduced 20 min after the addition of DFP, at which time there was a significant increase in ESA. (**b**) The ESA of three active electrodes from a representative slice. (**c**) Fold changes in mean ESA ± SEM after the washout of DFP and DHβE (from 60 to 80 min) relative to ESA in the presence of both DFP and DHβE (from 30 to 60 min). Each dot represents a single mouse. (**d**) Summary statistics of the data shown in panel (**c**) (N = 58 active electrodes from six independent slices from six mice).

We next tested the reversibility of post-DFP perfusion with DHβE (20 µM), which is a selective competitive antagonist of nAChRs that multimerizes with the α4 nAChR subunit. When added to the perfusate 20 min after DFP, a time during which DFP-increased ESA frequency was obvious and accelerating (Figure 5a,b), DHβE significantly dampened, stopped, or reversed the DFP-mediated acceleration of ESA frequency measured from active electrodes compared with DFP alone (compare Figure 4b to Figure 5b). DFP is an irreversible blocker of AChE, whereas DHβE is a reversible antagonist of the α4 nAChR. Figure 5b shows that the washout of both DFP and DHβE from the pMEA chamber for an additional 20 min quickly unmasked the irreversible nature of AChE inhibition by DFP, resulting in an escalating ESA frequency. Comparing the ESA frequencies recorded during perfusion with DFP and DHβE to those recorded after the washout revealed a 2.5 ± 0.4-fold increase in ESA frequency, likely due to the enduring actions of DFP in the absence of DHβE (Figure 5c,d).

#### 3.2.3. ESA Responses to DFP Are Significantly Attenuated in Slices from α4 nAChR Knockout (KO) Mice

**Figure 6:**
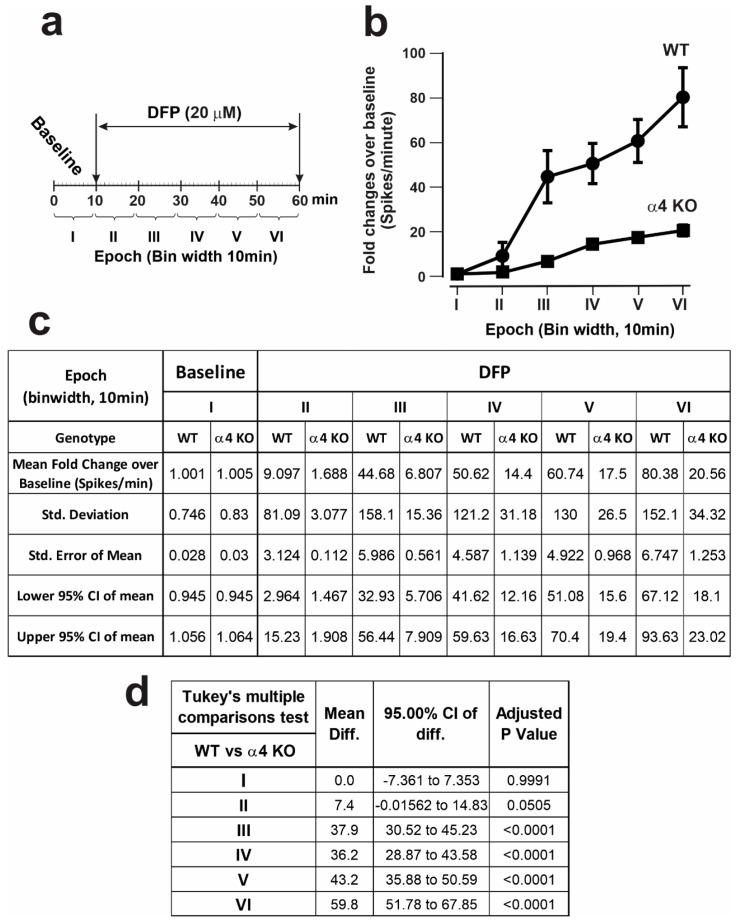
Greatly attenuated DFP responses in hippocampal slices from α4 nAChR KO mice. (**a**) Schematic of the experimental protocol in which DFP was added to hippocampal slices obtained from adult male wildtype (WT) or α4 nAChR KO mice. (**b**) The effect of genotype on DFP-triggered hyperactivity. Data are shown as the mean ESA ± 95% CI from Epochs II through VI, normalized according to the baseline (Epoch I). (**c**,**d**) The descriptive statistics and Tukey’s multiple comparison test results. Adjusted *p* values < 0.05 were regarded as significant. WT mice: N = 7, N = 10 slices, with a total N = 76 active electrodes. a4 KO mice: N = 6, N = 8 slices, with a total N = 68 active electrodes.

Among the nAChR subtypes, pentameric assemblies with two α4 subunits are the most abundantly expressed in the mammalian brain. Figure 6 shows that relative to the slices from wildtype (WT) mice, slices prepared from α4 nAChR KO mice displayed significantly attenuated DFP-triggered ESA responses. The epoch-wise comparisons of ESA frequencies showed significantly lower ESA frequencies across all epochs perfused with DFP in the α4 nAChR KO slices compared to the WT slices (Figure 6c,d).

### 3.3. Pharmacologic Antagonism or Genetic KO of α4, but Not α7, nAChRs Prevented DFP-Induced EEG Abnormalities

**Figure 7:**
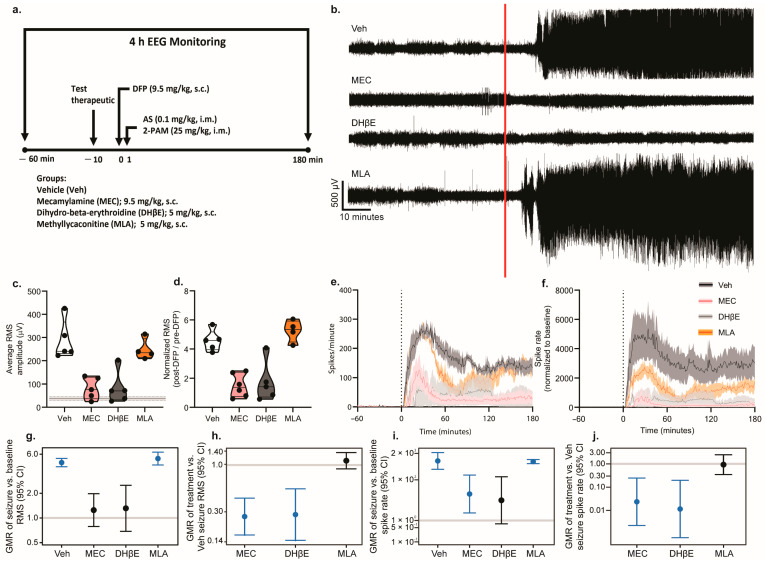
Pharmacologic antagonism of the α4 nAChR suppresses DFP-induced seizure activity. (**a**) Schematic illustrating the study design. Adult male C57/BL6J mice were instrumented for EEG monitoring of their seizure activity. On the day of experimentation, baseline EEG was recorded for 60 min prior to DFP intoxication. EEG monitoring continued for 180 min post DFP injection. Mice were pretreated with either a vehicle (Veh), the non-selective nAChR antagonist mecamylamine (MEC), the α4-selective nAChR antagonist dihydro-β-erythroidine hydrobromide (DHβE), or the α7-selective nAChR antagonist methyllycaconitine citrate (MLA) 10 min prior to the injection of DFP (9.5 mg/kg, s.c.), followed 1 min later by a combined injection of atropine sulfate (AS) (0.1 mg/kg, i.m.) and 2-pralidoxime (2-PAM) (25 mg/kg, i.m.). (**b**) Representative EEG traces for each experimental group. The red line indicates the time when DFP was administered. (**c**) Raw post-DFP RMS values in mice pretreated with either the Veh or one of the nAChR antagonists. Pre-DFP baseline values did not differ between groups and were therefore averaged across all groups and presented as a horizontal line ± SD. Data are presented as violin plots in which each point represents an individual animal, and the horizontal lines represent the minimum, quartile, median, and maximum values (*n* = 4–6 per group). (**d**) Post-DFP RMS values normalized to pre-DFP baseline RMS values in animals pretreated with the vehicle or one of the pharmacologic antagonists of nAChR (**e**) Raw EEG spike rates and (**f**) spike rates normalized to the baseline values over the recording period in the Veh (black) animals and animals treated with one of the pharmacologic antagonists of nAChR—MEC (pink), DHβE (gray), or MLA (orange). Data are presented as mean ± SEM (shaded areas) (*n* = 4–6 per group). The vertical dotted line indicates the time of DFP administration. (**g**) Geometric mean ratio (GMR) (dot) of the mean normalized early-seizure RMS value for each group relative to the pre-DFP baseline RMS with 95% confidence intervals (bars). The y-axis is shown as a log-scale. Confidence intervals that do not include 1 (the gray horizontal line) indicate a significant difference between the pre- and post-DFP RMS values. (**h**) GMR of the mean normalized RMS values for each group relative to the Veh-normalized RMS. (**i**) GMR of the mean change in spike rate for each group between the pre-DFP baseline and early-seizure periods. (**j**) GMR of the mean change in spike rate relative to the Veh group for each group between the pre-DFP baseline and early-seizure periods.

**Figure 8:**
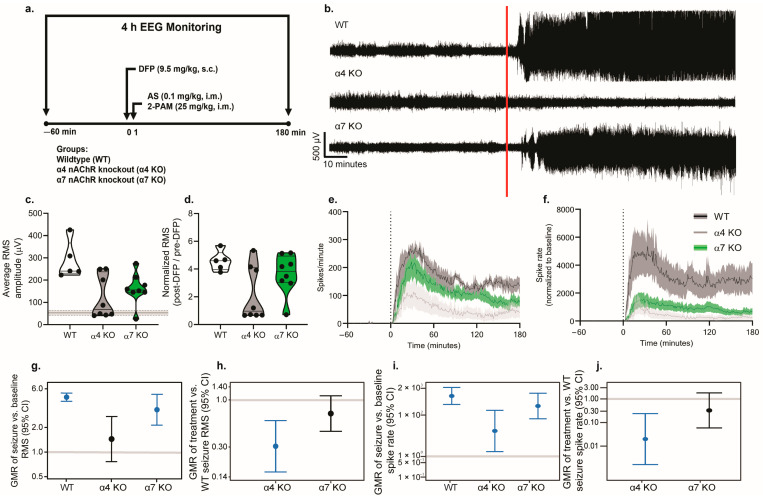
Genetic knockout of α4 nAChR attenuates DFP-induced seizures. (**a**) Schematic illustrating the study design. Adult male wildtype (WT), α4 nAChR knockout (KO), and α7 nAChR KO C57/BL6J mice were instrumented for EEG monitoring of their seizure activity. On the day of experimentation, baseline EEG was recorded for 60 min prior to DFP intoxication. EEG monitoring continued for 180 min post DFP injection. The WT animals are the same as the Veh animals presented in Figure 8 and were pretreated with a vehicle (PBS) 10 min prior to the injection of DFP. α4 and α7 KO animals did not receive any pretreatment. All animals were administered DFP (9.5 mg/kg, s.c.), followed 1 min later by a combined injection of atropine sulfate (AS) (0.1 mg/kg, i.m.) and 2-pralidoxime (2-PAM) (25 mg/kg, i.m.). (**b**) Representative EEG traces for each experimental group. The red line indicates when DFP was administered. (**c**) Raw post-DFP RMS values in WT, α4 KO, and α7 KO animals. Pre-DFP baseline values did not differ between groups and were therefore averaged across all groups and presented as the horizontal line ± SD. Data are presented as violin plots in which each point represents an individual animal, and the horizontal lines represent the minimum, quartile, median, and maximum values (*n* = 5–8 per group). (**d**) Post-DFP RMS values normalized to the pre-DFP baseline RMS in WT, α4, and α7 KO animals. (**e**) Raw EEG spike rates and (**f**) spike rates normalized to the baseline values over the recording period in WT (black), α4 KO (gray), and α7 KO (green) animals. Data are presented as mean ± SEM (shaded areas) (*n* = 5–8 per group). The vertical dotted line indicates the time of DFP administration. (**g**) Geometric mean ratio (GMR) (dot) of the mean normalized early-seizure RMS value for each group relative to the pre-DFP baseline RMS with 95% confidence intervals (bars). The y-axis is shown as a log-scale. Confidence intervals that do not include 1 (the gray horizontal line) indicate a significant difference between the pre- and post-DFP RMS values. (**h**) GMR of the mean normalized RMS values for each group relative to the WT-normalized RMS. (**i**) GMR of the mean change in spike rate for each group between the pre-DFP baseline and early-seizure periods. (**j**) GMR of the mean change in spike rate relative to the WT group for each group between the pre-DFP baseline and early-seizure periods.

To confirm the physiological relevance of our findings in acute hippocampal slices, we evaluated the effects of pharmacological antagonism or the genetic deletion of nAChRs on behavioral seizures and seizure activity in a mouse model of acute DFP intoxication [27]. In our initial assessments of DFP-induced seizure behavior, mice pretreated with either the nonspecific nAChR antagonist MEC or the α4-selective antagonist DHβE, and α4 KO mice, displayed attenuated seizure behavior compared to controls (Veh)/WT (Supplementary Figure S1b). Moreover, broad nAChR receptor antagonism significantly attenuated seizure behavior when administered 10 min, but not 40 min, after DFP administration (Supplementary Figure S1d). To confirm these behavioral data, subsequent experiments monitored seizure activity using EEG. Representative EEG traces demonstrate that acute intoxication with DFP significantly altered EEG activity in a manner consistent with seizure activity (Figure 7b and Figure 8b). Quantitative analyses of these data confirmed that acute DFP intoxication triggered electrographic seizure activity. Specifically, in animals pretreated with the vehicle treatment, both the EEG amplitude, as measured by the root-mean-squared (RMS) value, and the EEG spike rate were significantly increased within several minutes after DFP injection, and both metrics remained consistently elevated relative to pre-DFP baseline values for hours post DFP exposure (Figure 7).

Pharmacological antagonism (Figure 7) or genetic deletion (Figure 8) of the α4, but not the α7, nAChR prevented or significantly reduced DFP-induced seizure activity. WT mice pretreated with either the nonspecific nAChR antagonist MEC or the α4-selective antagonist DHβE (Figure 7c), and α4 KO mice (Figure 8c), exhibited post-DFP RMS values that were not significantly different from the pre-DFP baseline values. In contrast, raw RMS values were significantly elevated post DFP intoxication in WT mice pretreated with methyllycaconitine citrate (MLA), an α7-selective antagonist (Figure 7c), as well as in α7 KO mice (Figure 8c), and this relationship remained consistent after normalization of the RMS values to pre-DFP baseline values (Figure 7d and Figure 8d). Further analysis revealed that WT mice pretreated with the vehicle (geometric mean ratio [GMR] = 4.7, 95% confidence interval [CI] = 4.2–5.3, *p* < 0.001) or the α7-selective antagonist, MLA (GMR = 5.3, 95% CI = 4.4–6.3, *p* < 0.001) (Figure 7g), as well as the α7 KO mice (GMR = 3.4, 95% CI = 2.2–5.2, *p* < 0.001) (Figure 8g), had RMS values during the early-seizure period that were three to five times higher, on average, than their baseline values.

Raw and normalized EEG spike rates following DFP intoxication were also differentially altered through the pharmacological (Figure 7e,f), but not genetic (Figure 8e,f), manipulation of nAChR signaling. All pharmacological treatment groups other than the DHβE-pretreated WT mice exhibited a significant increase in their spike rates during the first hour post DFP exposure relative to baseline values (Veh/WT: GMR = 852.3, 95% CI: 330.1–2200.6, *p* < 0.001; MEC: GMR = 20.1, 95% CI = 2.4–171.4, *p* = 0.007; MLA: GMR = 788.8, 95% CI = 625.0–995.5, *p* < 0.001) (Figure 7i). Similarly, both genetic KO groups displayed significantly elevated spike rates during the first hour post intoxication relative to baseline values (α4 KO: GMR = 17.0, 95% CI = 1.7–169.2, *p* = 0.02; α7 KO: GMR = 277.0, 95% CI = 66.6–1153.0, *p* < 0.001) (Figure 8i).

We next compared the degree of electrographic responses to acute DFP intoxication between the experimental groups to determine which groups differed significantly from WT mice intoxicated with DFP in the absence of any pharmacological or genetic manipulation. The WT mice pretreated with either MEC (GMR = 0.3, 95% CI = 0.2–0.4, *p* < 0.001) or DHβE (GMR = 0.3, 95% CI = 0.1–0.5, *p* < 0.001) (Figure 7h) and the α4 KO (GMR = 0.3, 95% CI = 0.2–0.6, *p* < 0.001) mice (Figure 8h) exhibited a 70% reduction in the post-DFP increase in RMS compared to Veh/WT controls. We did not observe significant differences between normalized RMS values in the mice pretreated with MLA (Figure 7h) or α7 KO mice (Figure 8h) relative to the Veh/WT controls.

The change in spike rate between the baseline and post-DFP time points for each experimental group was also compared to the Veh/WT controls intoxicated with DFP. The difference in spike rate between the baseline and early-seizure periods was significantly reduced in WT mice pretreated with MEC (GMR = 0.02, 95% CI = 0.002–0.25, *p* = 0.002) or DHβE (GMR = 0.01, 95% CI = 0.001–0.20, *p* = 0.002) (Figure 7j) and in α4 KO (GMR = 0.02, 95% CI = 0.002–0.24, *p* = 0.002) mice (Figure 8j) compared to the Veh/WT controls. No significant differences in the rate of DFP-induced spiking were detected between the controls and WT mice pretreated with MLA (Figure 7j) or α7 KO animals (Figure 8j).

Additionally, we evaluated weight loss following acute OP intoxication as a predictor of functional recovery [42]. Across all experimental groups, there were no significant differences in body weight loss at either 4 or 24 h following DFP intoxication (Supplementary Figure S2).

## 4. Discussion

Previous studies have demonstrated that non-specific nicotinic antagonism suppresses parasympathetic symptoms and reduces mortality following acute OP intoxication [43,44]. While α4 [45,46] and α7 [30,47] nAChRs modulate neuronal excitability, their involvement in OP-induced seizures has yet to be robustly investigated. Using complementary pharmacologic and genetic approaches in acute hippocampal slices prepared from adult mice and an in vivo seizure mouse model, we found that α4, but not α7, nAChR signaling is involved in the induction of electrical spike activity and seizure activity following exposure to high levels of OPs.

Using the mouse hippocampal slice preparation experiments, we found that the inhibition of nAChRs with the non-selective nAChR antagonist MEC or the α4-selective nAChR antagonist DHβE significantly attenuated DFP-triggered ESA when added either before (MEC) or within 10 min after DFP addition. DFP-induced increases in ESA frequency were also attenuated in hippocampal slices prepared from α4 KO animals relative to WT controls. These in vitro observations were predictive of in vivo effects in that behavioral and electrographic metrics of DFP-induced seizure activity were significantly reduced through pretreatment with MEC or DHβE, or genetic deletion of the α4 nAChR subunit.

Our findings address the limited data regarding the role of nAChRs in models of acute OP intoxication. While early in vivo pharmacologic work emphasized the role of the mAChR in OP-induced seizures, nAChR-sensitive soman-induced seizures have been reported earlier in the presence of a high, anti-convulsant dose of atropine sulfate [22]. Such data hinted at a role for nAChR signaling in OP-induced seizures, but the lack of appropriate experimental tools complicated subsequent attempts to parse out the relative contribution of nAChRs vs. mAChRs in OP-induced seizures. The in vivo model we used in our studies provided a low, peripherally active dose of atropine sulfate to improve survival without robust central effects [48]. Using this model, we were able to demonstrate the role of nAChRs in OP-induced seizures independent of central mAChR antagonism.

The limited data available regarding the involvement of specific nAChR subtypes in models of acute OP intoxication has been due in part to the lack of cell culture models that appropriately target nAChRs to the neuronal cell surface, and thus fail to respond to stimuli that activate nAChR signaling [25,26]. The use of hippocampal slices prepared from adult mice overcame this obstacle as shown by their responsiveness to DFP. We observed delayed AChE inhibition in these preparations, which we think reflects the slow kinetics of DFP accessing the catalytic pocket of AChE. This delay is consistent with the significantly slower inhibition kinetics of AChE, which resulted in K_M_ and K_i_ measures being order of magnitudes higher than those of other fluorophosphate nerve agents such as soman and sarin [49]. As would be expected, the slow rate of AChE inhibition in the slice preparation remains dependent on the DFP concentration in the perfusate, as shown in Figure 2, and is consistent with temporal delays in DFP effects on slice activity [50]. An important observation is that increases in ESA firing are not only temporally determined by the DFP concentration but are tightly tied to the temporal suppression of AChE activity, requiring a substantial fraction of AChE suppression before significantly increased ESA is observed. It should be noted that ESA rates observed in hippocampal slices may also depend on consequential increases in the glutaminergic signaling that regulates the excitability of the hippocampal slice [37].

Here, we leveraged DFP-responsive in vitro hippocampal slices and nAChR antagonists with differential subunit specificities to demonstrate that α4, but not α7, nAChRs significantly contributed to acute OP-induced hyperexcitability. Further, these observations were confirmed in vivo. While the role of the α4 nAChR subtype in OP-induced seizures has not been previously investigated, earlier studies have identified the insensitivity of DFP-associated seizures to α7 nAChR signaling [51], and the inefficacy of delayed broad nAChR antagonism or α7-selective antagonism in reducing soman-induced spiking in hippocampal slice preparations [52]. Our findings, paired with these observations, confirm the α7-independence of OP-induced spiking. Additionally, the ineffectiveness of delayed nAChR antagonism is consistent with the timeline of the cholinergic–glutamatergic transition required for the maintenance of OP-induced seizures [21].

In non-OP models, it is established that nAChR activation can induce seizures; however, the role of receptor subtypes in seizure generation is not well understood. Nicotine-induced seizures are blocked by broad nAChR antagonism [53], but there are discrepant reports regarding the involvement of α4 vs. α7 nAChRs. Peripheral and central administrations of α7-selective antagonists, including MLA or α-nudicauline, were shown to attenuate nicotine-induced seizures, but because their mitigating effects were significantly less potent than those produced by MEC and occurred in a dose range known to affect nicotinic responses not mediated by α7, the authors concluded that the involvement of α4β2 receptor subtypes could not be ruled out [53]. The authors also concluded that the α7 nAChR subtype does not play a major role in initiating nicotine-induced seizures, a conclusion that is consistent with our observations of the lack of effect on DFP-induced seizures of pharmacological antagonism or genetic deletion of the α7 nAChR. Homozygous α5 nAChR subunit KO mice are highly resistant to nicotine-triggered seizures in the presence or absence of the β4 nAChR subunit [54,55], whereas a genetic model of α4 hypersensitivity exhibited increased susceptibility to nicotine-induced seizures [46]. Previous studies of α4 nAChR subunit KO mice indicated that compared to WT mice, they did not exhibit spontaneous seizures but did exhibit heightened anxiety and an altered time course in developing behavioral topographies in response to non-seizurogenic doses of nicotine [29]. Furthermore, α4 nAChR subunit KO mice had a greater sensitivity to the GABA receptor blockers, pentylenetetrazol and bicuculline, and to a lesser extent, the glutamate receptor agonist kainate [56]. In contrast, α4 KO mice were significantly protected against 4-aminopyridine-induced major motor seizures and death compared to WT mice [56]. Collectively, these results point to the importance of the α4 and α5 nAChR subtypes in modulating sensitivity to seizure induction by nicotine. However, whether either subtype is essential for OP-triggered seizure induction, which is characterized by elevated synaptic acetylcholine (ACh) [57], has not been directly tested. While ACh is known to mediate seizure generation in epileptic models [58,59], this relationship has not been comprehensively evaluated in naïve animals. Similarly, in slice preparations, exogenous ACh exerted excitatory effects [60,61,62], but the capacity for excessive synaptic ACh to induce seizures and the role of particular AChR subtypes in ictal activity has not been rigorously investigated. Our findings address this data gap, providing the first definitive evidence that α4 nAChR subtypes are necessary for neuronal hyperexcitability induced by cholinergic receptor hyperstimulation, and are involved in OP-induced seizures in vivo.

We observed that α4 antagonism post DFP incubation attenuated and occasionally reversed DFP-triggered electrical spike hyperactivity in individual hippocampal slices, possibly the result of the rapid engagement of additional receptor subtypes or neurotransmitter systems post OP exposure. Our in vivo observation that MEC administered 10 min, but not 40 min, post DFP exposure attenuated seizure activity (Supplementary Figure S1d) further suggested the rapid activation of non-α4 nAChR subunits following acute OP intoxication. The ability of MEC to inhibit seizure activity when administered shortly after DFP exposure is consistent with reports stating that glutamatergic systems are engaged within 5 min of acute OP intoxication [21], and that high concentrations of MEC (~100 µM) inhibit NMDA-mediated currents [63]. It has previously been demonstrated that MEC administered at 5 mg/kg, i.v., resulted in a brain concentration of MEC of ~42 µM [64]; thus, it is possible that our dosing paradigm achieved brain concentrations of MEC high enough to inhibit both nAChRs and NMDA receptors. Additional investigations are needed to fully resolve the temporal relationship between acute OP intoxication and the activation of distinct neurotransmitter systems/receptor subtypes in the brain.

As can be expected with intrinsically different models (acute hippocampal slice preparations vs. in vivo cortical EEG monitoring), there was some quantitative disparity in the effects of pharmacologic and genetic modulation of nAChR signaling on the response to DFP. For example, we observed more pronounced suppression of DFP-induced seizure activity/hyperexcitability in vivo compared to in the slice preparations. Previous publications have described the necessity of an antimuscarinic for the efficacy of nAChR antagonism against non-electrographic aspects of OP-associated toxicity [65,66], suggesting that our in vivo use of atropine sulfate and 2-pralidoxime may influence the response to DFP exposure. Additionally, OP-induced seizures are thought to originate external to the hippocampus [21], and while acute hippocampal slice preparations replicate many features of the intact nervous system, they lack afferent input [67]. Such differences in the circuitry composition and/or degree of circuit engagement in hippocampal slice spike activity versus in vivo cortical EEG monitoring may underlie the differences in our models.

In vivo seizure monitoring revealed a bimodal effect of α4 nAChR KO on seizure induction. While the majority of α4 nAChR KO animals did not display the electrophysiologic shifts characteristic of a seizure following acute DFP intoxication, the initial response of a subpopulation of the KO animals to DFP was similar to that of the WT animals. The same bimodal distribution was not seen with pharmacologic inhibition of the α4 nAChR. Similarly, we observed incomplete inhibition of DFP-induced ESA responses in hippocampal slices prepared from α4 nAChR KO mice. There is limited evidence that global α4 KO produces compensatory shifts in cholinergic signaling [68], which could variably influence responses to acute OP intoxication. Pharmacospecificity may underlie the differences in responses to DHβE treatment and α4 KO; DHβE has been shown to inhibit α3 nAChRs under certain conditions [69]. While our data suggest that α4 signaling is a primary driver of OP-induced seizures, α3 nAChRs are known to contribute to nicotine-associated seizures [70]. It is possible that α3 nAChRs may be involved in OP-induced electrical spiking, although to a limited degree. The involvement of additional receptor subtypes in OP-induced spiking is further suggested by our observation of significantly attenuated, but still elevated, electrical spike rates immediately following DFP intoxication in α4 KO animals compared to WT animals. Thus, while our pharmacologic data demonstrate clear evidence that α4 nAChR signaling is involved in DFP-induced seizures, α4 KO does not prevent all OP-associated hyperexcitability. It is likely that incomplete suppression of DFP-induced spiking in the α4 KO animals underlies the limited, but still evident, behavioral seizures in these animals (Supplementary Figure S1c). Additional investigations will be needed to clearly define the role of α3 and additional nAChRs in OP-mediated seizures.

Our findings are important for advancing the development of anti-seizure therapeutics, particularly prophylactics for individuals at high risk of acute OP intoxication. Historically, such individuals have received pyridostigmine bromide (PB), a reversible AChE inhibitor, to mitigate OP-associated toxicity. However, PB use has been tied to a number of adverse health outcomes [71,72], highlighting the need for prophylactic alternatives. While we observed comparable anti-seizure efficacy with broad nAChR and α4-selective antagonism, non-selective nAChR inhibitor ganglionic blockers can produce concerning autonomic consequences [73]. In contrast, our data identified α4 nAChRs as a feasible target for future prophylactic development, warranting scientific investment.

## 5. Conclusions

In summary, we offer compelling evidence for the necessary role of α4 nAChR activation in seizure induction following acute OP intoxication. We demonstrated that OP-induced seizures are sensitive to genetic or pharmacologic inhibition of α4, but not α7, nAChRs and that these observations are consistent between in vitro and in vivo assessments. These findings advance our understanding of the nicotinic cholinergic system in seizure activity and have implications for the development of therapeutics.

## Figures and Tables

**Table 1:** Primer sequences used to genotype the transgenic animals used in this study.

Gene Name	Forward Primer Sequence	Reverse Primer Sequence	Expected Size (bp)
α4-WT	5′–CAATGTACACCACCGCTCAC–3′	5′-ACTGCTATTGGGTGGGTGAC–3′	385
α4-deletion mutant	5′–CTTGGGTGGAGAGGCTATTC–3′	5′–AGGTGAGATGACAGGAGATC–3′	280
α7-WT	5′–TTCCTGGTCCTGCTGTGTTA–3′	5′–ATCAGATGTTGCTGGCATGA–3′	390
α7-deletion mutant	5′–TTCCTGGTCCTGCTGTGTTA–3′	5′–CCCTTTATAGATTCGCCCTTG–3′	187

**Table 2:** Common neuronal cell culture models express AChE but do not respond to OPs (DFP or paraoxon) or nicotine with altered spontaneous calcium oscillations (SCOs).

Primary Neuron-glia c¥o-cultures (Age When Cells were Derived)	SCO Response to DFP with >95% AChE Inhibition	AChE Expressed	Response to Cholinergic Ligands	
			Atropine	Nicotine
**Murine**				
**Hippocampus (PND 0)**	DFP **(−)**; Paraoxon **(−)**	**YES**	**YES**	**NO**
**Neocortex (PND 0)**	DFP **(−)**; Paraoxon **(−)**	**YES**	**YES**	**NO**
**Neocortex (E 18)**	DFP **(−)**; Paraoxon **(−)**	**LOW**	(Not tested)	**NO**
**Neocortex (adult)**	Unhealthy	(Not tested)	(Not tested)	(Not tested)
**Rat**				
**Hippocampus (PND 0)**	DFP **(−)**; Paraoxon **(−)**	**YES**	**YES**	**NO**
**Neocortex (PND 0)**	DFP **(−)**; Paraoxon **(−)**	**YES**	(Not tested)	**NO**

AChE = acetylcholinesterase; DFP = diisopropylfluorophosphate; E = embryonic day; PND = postnatal day; SCO = spontaneous calcium oscillation.

**Table 3:** Statistical analyses of electrical spike activity (ESA; spikes/minute), represented as fold changes over the baseline values of the same electrode (CI = confidence interval).

	Baseline	DFP				
**Epoch (bin width, 10min)**	**I**	**II**	**III**	**IV**	**V**	**VI**
**Mean fold-change over baseline (spikes/min)**	1	9.1	44.7	50.6	60.7	80.4
**Std. deviation**	0.746	81.1	158	121	130	152
**Std. error of mean**	0.0282	3.12	5.99	4.59	4.92	6.75
**Lower 95% CI of mean**	0.945	2.96	32.9	41.6	51.1	67.1
**Upper 95% CI of mean**	1.06	15.2	56.4	59.6	70.4	93.6
**Dunnett’s Multiple Comparisons Test**	**Mean Diff.**		**95% CI of diff.**		**Adjusted *p* Value**	
**I vs. II**	−59.33		−76.65 to −42.01		<0.0001	
**I vs. III**	−248.4		−294.5 to −202.1		<0.0001	
**I vs. IV**	−408.6		−462.1 to −355.0		<0.0001	
**I vs. V**	−542.3		−604.0 to −480.6		<0.0001	
**I vs. VI**	−678.9		−760.0 to −597.7		<0.0001	

## Data Availability

The raw data supporting the conclusions of this article will be made available from the authors on request.

## Supplementary Materials

The following supporting information can be downloaded at https://www.mdpi.com/article/10.3390/toxics12040263/s1, Genotyping details; Figure S1: Behavioral seizure scores of DFP-intoxicated animals with or without pharmacological antagonism or genetic knockout of nAChR; Figure S2: Percent body weight loss in DFP-intoxicated animals with or without pharmacological antagonism of genetic knockout of nAChR.
