# Supplementary material for: The α4 Nicotinic Acetylcholine Receptor Is Necessary for the Initiation of Organophosphate-Induced Neuronal Hyperexcitability
Source: Toxics. 2024 Mar 31;12(4):263. doi: 10.3390/toxics12040263 (PMC11054284; doi:10.3390/toxics12040263)
Supplement: Supplementary file 1 [file toxics-12-00263-s001.zip › toxics-2897054-supplementary.pdf]

Supplemental Materials

The α4 Nicotinic Acetylcholine Receptor Is Necessary for the Initiation of Organophosphate-Induced Neuronal Hyperexcitability

Peter M. Andrew [1,†], Wei Feng [1,†], Jonas J. Calsbeek [1,†], Shane P. Antrobus [1], Gennady A. Cherednychenko [1], Jeremy A. MacMahon [1], Pedro N. Bernardino [1], Xiuzhen Liu [1], Danielle J. Harvey [2], Pamela J. Lein [1,*] and Isaac N. Pessah [1,*]

α4/ α7 genotyping protocol details:

Reaction Mix:

**Reagent**	**Volume (µl)**
DNA Sample	2
Go Taq Green, Promega #M7123	12.5
Primer 1 – stock concentration 50 µM	0.25
Primer 2 – stock concentration 50 µM	0.25
Primer 3 – stock concentration 50 µM	0.25
Primer 4 – stock concentration 50 µM	0.25
Double-distilled H_2O	9.5

Thermocycler Program:

**Steps**	**Temp (°C)**	**Time**	**# of Cycles**
1. Initiation/Melting	94	3 min	1
2. Denaturation	94	45 sec	1
3. Annealing	60	30 sec	1
4. Elongation	72	1 min	1 – return to step 2 x 35 iterations
5. Amplification	72	10 min	1
6. Finish	4	n/a	n/a

a.

Score	Behavior
5	Falling with loss of righting reflex
4	Rearing, falling, tonic extensions
3	Forelimb or hindlimb clonus
2	Muscle tremors, head nod
1	Straub tail, SLUD
0	No abnormal symptoms

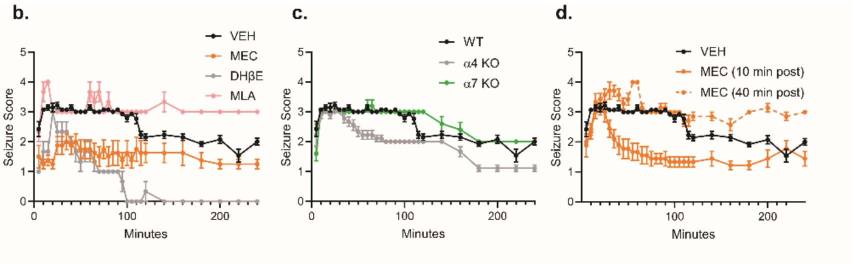

Supplemental Figure S1. (a) Behavioral scoring scale used to evaluate seizures following DFP administration. SLUD = salivation, lacrimation, urination, defecation. (b) Average behavioral seizure scores over time following acute DFP intoxication. Mice were pretreated with vehicle (VEH), the nonselective nAChR antagonist mecamylamine (MEC), the α4-selective nAChR antagonist dihydro-β-erythroidine hydrobromide (DHβE), or the α7-selective nAChR antagonist methyllycaconitine citrate (MLA) 10 min prior to injection of DFP (9.5 mg/kg, s.c.), followed 1 min later by a combined injection of atropine sulfate (AS) (0.1 mg/kg, i.m.) and 2-pralidoxime (2-PAM) (25 mg/kg, i.m.). Data are presented as mean ± SEM (n= 3 – 14). (c) Average behavioral seizure score over time following DFP intoxication in wildtype (WT), α4 nAChR knockout (α4 KO), and α7 nAChR knockout (α7 KO) animals. Data are presented as mean ± SEM (n= 5 – 14). WT animals are the same as VEH animals presented in panel (b). (d) Average behavioral seizure score over time following DFP intoxication in VEH animals (same animals as shown in panels b and c) or mice treated with MEC 10- or 40-min post-DFP intoxication. Data are presented as mean ± SEM (n= 9-14).

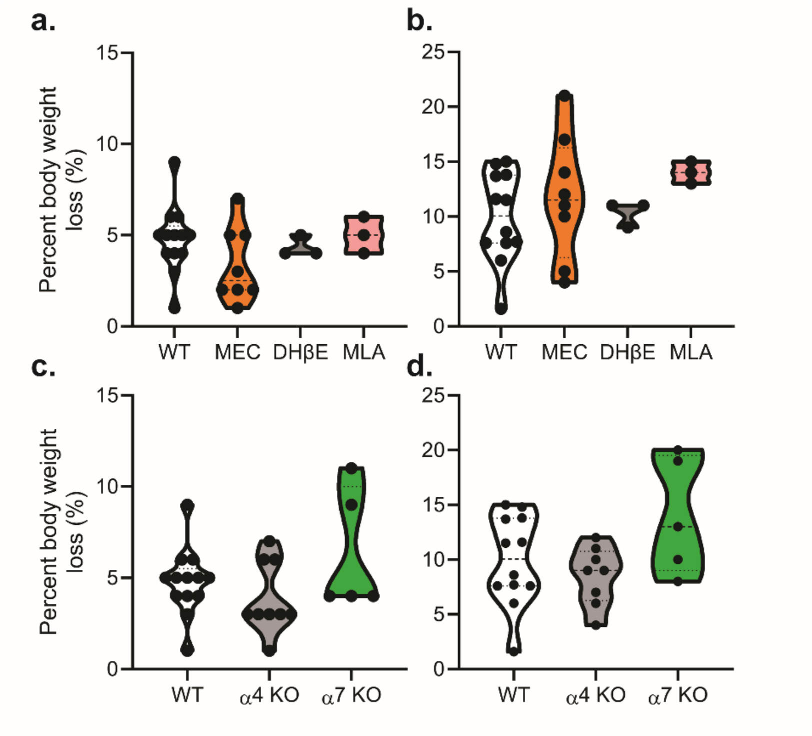

Supplemental Figure S2. (a, b) The impact of pretreatment with nAChR antagonists on body weight loss at 4 (a) and 24 hours (b) post-DFP intoxication. (c, d) Percent body weight loss at 4 (c) and 24 hours (d) post-DFP intoxication in WT, α4 KO, and α7 KO animals. Data are presented as violin plots in which each point represents an individual animal, and the horizontal lines represent the minimum, quartile, median, and maximum values (n= 3 – 14). WT animals are the same as VEH animals presented in panels (a) and (b).
